# Some Queries

**Published:** 1883-11

**Authors:** 


### SOME QUERIES.

Dear Editor:—As it is much easier to ask questions than it is to answer them, it is only "too utterly" human to fall into doing the easiest thing.

When it is exacted that a dose of homœopathic medicine shall be a tangible one, what sense is it expected to gratify in order that it be such?

As drugs are proven singly, and there is no law to guide in alternation, how are those who use more than one remedy at a time to be instructed and strengthened in their experience?

What benefits are likely to accrue from the recent change in the ancient code of the Allopathic Society? Will oil and water mix today any better than it ever did?

At what stage in a case will it be advisable to call in allopathic counsel?

Which is the hero in "heroic treatment," the doctor or the patient? [Patient; *vide* Garfield.—Ed.]

Will those who, with microscopic lens, are constantly trying to pick flaws in Hahnemann's theory, show us a better and more successful way? Or will they continue to find fault with the water because they can't swim?

If the dynamists sometimes fail, how can the materialists and eclectics ever hope to succeed?

Will those who believe in the partial application of the homœopathic law tell us just where order fails and confusion wins?

Is giving the large spoonful oft repeated of the strongly smelling, darkly looking, pungent tasting substances from two, three, or five glasses in rotation the "homœopathic pura" so many are clamoring for?

Why do the materialists continue fraternal relations with the etherial dynamists if they are such a despisable sect? Can it be that the air of restful certainty, assured success, and patient confidence surrounding the latter is equally as magnetic as it is annoying?

Seeing that the proving of drugs is not pursued to the killing point, how can the existing pathological condition of a patient be positively determined?

